# Pneumonia Risk and Use of Angiotensin-Converting Enzyme Inhibitors and Angiotensin II Receptor Blockers

**DOI:** 10.2188/jea.JE20120112

**Published:** 2013-09-05

**Authors:** Chia-Lin Liu, Wen-Yi Shau, Chia-Hsuin Chang, Chi-Shin Wu, Mei-Shu Lai

**Affiliations:** 1 Department of Family Medicine, En Chu Kong Hospital, New Taipei City, Taiwan; 2 Graduate Institute of Epidemiology and Preventive Medicine, College of Public Health, National Taiwan University, Taipei, Taiwan; 3 Division of Health Technology Assessment, Center for Drug Evaluation, Taiwan, Taipei, Taiwan; 4 Graduate Institute of Clinical Medicine, College of Medicine, National Taiwan University, Taipei, Taiwan; 5 Department of Internal Medicine, National Taiwan University Hospital, Taipei, Taiwan; 6 Department of Psychiatry, Far Eastern Memorial Hospital, New Taipei City, Taiwan

**Keywords:** angiotensin-converting enzyme inhibitor, angiotensin II receptor blockers, pneumonia, case-crossover study

## Abstract

**Background:**

Recent studies have shown that use of angiotensin-converting enzyme (ACE) inhibitors may decrease pneumonia risk in various populations. We investigated the effect of ACE inhibitors and angiotensin II receptor blockers (ARBs) on pneumonia hospitalization in the general population of Taiwan.

**Methods:**

We conducted a case-crossover study using the Taiwan Longitudinal Health Insurance Database for the year 2005. Data from patients hospitalized for the first time for pneumonia during 1997–2007 were analyzed. The case period was defined as the 30 days before admission; the periods 90 to 120 days and 180 to 210 days before admission were used as control periods. Prescribing status of ACE inhibitors and ARBs during the 3 periods was assessed for each patient. Conditional logistic regression was used to estimate the odds ratio (OR) for pneumonia associated with use of ACE inhibitors and ARBs.

**Results:**

We identified 10 990 cases of hospitalization for new pneumonia. After adjustment for time-variant confounding factors, pneumonia was not associated with use of ACEI or ARBS: the ORs were 0.99 (95% CI, 0.81–1.21) and 0.96 (0.72–1.28), respectively. No association was seen for cumulative defined daily doses (DDDs), as compared with nonusers, for 0 to 30, 31 to 60, or more than 60 DDDs. The results were found to be robust in sensitivity analysis.

**Conclusions:**

Neither the use nor cumulative dose of ACE inhibitors or ARBs was associated with pneumonia among the Taiwanese general population.

## INTRODUCTION

Pneumonia is a frequent cause of death due to infectious disease.^1^ Silent aspiration is a predisposing factor for pneumonia^2^ and is prevalent among elderly patients with impaired cough reflex.^3^ Angiotensin-converting enzyme (ACE) inhibitors induce a cough reflex by blocking degradation of substance P. A previous study showed that stroke patients receiving ACE inhibitors had higher levels of substance P and improvement in silent aspiration.^4^^,^^5^ Many studies have shown that ACE inhibitors prevent pneumonia risk in stroke patients, especially among Asian patients.^6^^–^^12^ The effects of ACE inhibitors have been investigated in other populations, but the results were inconsistent.^13^^–^^16^

In addition to the effects of ethnicity and the underlying risk factors for pneumonia, previous studies were limited by methodologic issues. Several confounding factors were not adequately adjusted for, such as smoking, impaired functional and cognitive status, and unhealthy lifestyle, which are risk factors for pneumonia. The inconsistent findings of those studies might have been due to these unmeasured confounding factors.

In this study, we investigated the effects of ACE inhibitors and angiotensin II receptor blockers (ARBs) on pneumonia risk among the general population of Taiwan, a population susceptible to ACE-inhibitor induced cough.^17^^,^^18^ In addition, we compared the effects of ACE inhibitors and ARBs on pneumonia risk and tested the effect of ACE inhibitors and ARBs on pneumonia risk among patients with different underlying illnesses.

## METHODS

### Data source

We used the Longitudinal Health Insurance Database for the year 2005 (LHID2005) in the present study. The National Health Insurance (NHI) covered more than 98% of the population of Taiwan. The NHI claims database for academic research purposes is prepared by the Taiwan National Health Research Institute (NHRI) and contains detailed information on each outpatient prescription, including drug name, dosage, fee, prescribing date, and duration of prescription. It also contains inpatient information, including date and duration of hospitalization, and up to 5 discharge diagnosis codes, which were coded according to the International Classification of Diseases, Ninth Revision, Clinical Modification (ICD-9 CM). The LHID2005 contains all claims data for 1 million beneficiaries, who were randomly sampled from approximately 25.6 million registered beneficiaries of the NHI in year 2005. There was no significant difference in the distributions of sex, age, or average insured payroll-related amount between patients in the LHID2005 and the original population.^19^ To comply with privacy regulations regarding personal electronic data, personal identities were encrypted, and all data were analyzed anonymously. The study protocol was approved by the research ethics committee of National Taiwan University Hospital (201002042R).

### Patients

The study period was the 11-year period from January 1, 1997 through December 31, 2007. We identified cases using ICD9-CM codes from the 5 discharge diagnosis codes from all hospitalizations during the study period. Cases were selected according to the following criteria: an adult (age >18 yrs) hospitalized with a first discharge diagnosis of pneumonia (480–483, 485–487) or aspiration pneumonia (507), or a patient with a secondary diagnosis of pneumonia (480–483, 485–487) in combination with a primary diagnosis of respiratory failure (518.8) or sepsis (038).^20^ Patients were excluded if they had less than 1 year of continuous records in the NHI before developing pneumonia or if their record had no information on sex. To increase the stability of the health status of the study population, we excluded patients hospitalized for any cause within 1 year before a pneumonia diagnosis. For a person with multiple pneumonia diagnoses, only the first episode was included.

### Study design

We used a case-crossover design to assess the effects of ACE inhibitors and ARBs on pneumonia hospitalization. The case-crossover design uses within-subject comparisons of drug exposures over time to estimate the rate ratio of the outcome association with the drug under study. Because of the use of within-subject comparison, the case-crossover design auto-adjusts for unmeasured time-invariant confounding factors among different patients.^21^^,^^22^ This design has proven very useful in recent pharmacoepidemiologic studies.^23^^,^^24^

Exposure to ACE inhibitors and ARBs during the case period was compared with exposure status during the control periods for each patient. ACE inhibitors and ARBs were frequently prescribed to hypertensive patients. Physicians in Taiwan usually prescribe medicine for 28 or 30 days in 1 prescription. Hence, we defined the case period as the 30-day period immediately before the admission date, which was the defined as the index date for pneumonia hospitalization. The control periods were the periods 91 to 120 days and 181 to 210 days before the index date. We also directly compared the effects of ACE inhibitors and ARBs on pneumonia risk.

Because some patients may have received treatment in an outpatient clinic before admission, we conducted a sensitivity analysis by moving the index date to 14 days before admission. To evaluate the robustness of the study results, we performed additional sensitivity analyses by altering the length of each time period to 14 days, 60 days, and 75 days, as not all patients took medication regularly. They may have taken their remaining medication and delayed refilling of prescriptions, which could result in exposure misclassification. Hence, we maintained a minimum washout period of 2 weeks between the case and control periods and limited the maximum exposure period to 75 days.

### Variable assessment

The prescribing status of ACE inhibitors and ARBs during the case and control periods was assessed for each patient. We identified the drugs by Anatomical Therapeutic Chemical (ATC) code (ACE inhibitors: C09A, C09B; ARBs: C09C, C09D). During each period, we calculated the cumulative prescribed dose, using defined daily dose (DDD), which is the assumed average maintenance dose per day for a drug used for its main indication in adults.^25^ In analyses of the dose–response effect of each drug on pneumonia risk, the cumulative dose was categorized into 3 ranges: 0 to 30, 31 to 60, and more than 60 DDDs.

We also collected information on patient age, sex, other medication used, number of outpatient clinic visits, and comorbidities. Previous studies showed statin (ATC code: C10AA, C10BA, C10BX) use decreased pneumonia risk by means of an immunomodulating effect.^16^^,^^26^ Proton-pump inhibitors (PPIs, ATC code A02BC) and H2 blockers (ATC code A02BA) were reported to increase pneumonia risk,^27^ possibly because reduced secretion of gastric acid allows pathogen colonization from the upper gastrointestinal tract, thereby resulting in pneumonia via aspiration. In addition, the general condition of patients could affect the number of visits to physicians. Hence, exposures to statins, PPIs, and H2 blockers and number of outpatient clinic visits during each period were included as time-variant confounding factors in the adjusted analyses.

A past history of stroke was defined as any discharge code for stroke (ICD-9 CM 430–434) before pneumonia. Diabetes was defined as use of any antidiabetes drug (ATC code: A10) during the case or control periods.

### Statistical analysis

We calculated odds ratios (ORs) and 95% CIs for pneumonia associated with use versus nonuse of ACE inhibitors and ARBs. Conditional logistic regression was used because of the presence of paired data (1 case period matched to 2 control periods). We calculated crude ORs for pneumonia associated with ACE inhibitors and ARBs, and ORs adjusted for exposure (yes/no) to statins, PPIs, and H2 blockers during each period. We also adjusted for number of outpatient clinic visits during each period. Additionally, we performed subgroup analyses of patients with stroke, patients with diabetes, and people aged 65 years or older. A 2-sided *P* value of less than 0.05 was considered to indicate statistical significance. All statistical calculations were performed using commercially available software (SAS version 9.1.3, Cary, NC, USA).

## RESULTS

A total of 10 990 cases of pneumonia requiring hospitalization were identified for analysis. The baseline characteristics of the patients are shown in Table 1. The study population had a mean age of 57.6 ± 20.5 years, and 45% of patients were women. Less than 5% of the study population had a history of stroke, and nearly 44% were aged 65 years or older. Overall, 1277 patients used diabetes medications, 1030 used ACE inhibitors, and 638 used ARBs during the case or control periods.

**Table 1.** Patient demographic and clinical characteristics, *n* = 10 990

Variables	Number or mean	% or SD
Female	4890	(44.49)
Age, yrs	57.64	(20.56)
Hospitalization >21 days^a^	563	(5.20)
Stroke	527	(4.80)
Diabetes	1277	(11.62)
Age ≥65 yrs	4816	(43.82)
ACE inhibitor user	1030	(9.37)
ARB user	638	(5.81)

Abbreviations: ACE, angiotensin-converting enzyme; ARB, angiotensin II receptor blocker.

^a^Data missing for 158 patients.

In the present study, we were more interested in community-acquired pneumonia (CAP) than in nosocomial pneumonia; however, these conditions are difficult to distinguish in a claims database. In Taiwan, physicians would be more likely to code pneumonia as a primary or secondary discharge diagnosis if the patient had been admitted for CAP and to code pneumonia as a latter diagnosis if the patient had developed nosocomial pneumonia. In addition, most patients with CAP would be hospitalized for less than 21 days in Taiwan, and most cases of nosocomial pneumonia would result in prolonged hospitalization. Hence, we examined duration of hospitalization in our study population. In most cases, duration of hospitalized was shorter than 21 days. Thus, we assumed that most of the study patients had CAP. Although 158 of the 10 990 patients had missing data for duration of hospitalization, this proportion is too low (approximately 1.4%) to affect the final results.

Associations of ACE inhibitors and ARBs with pneumonia are shown in Table 2. After adjustment for time-variant confounding factors, pneumonia was not associated with use of ACE inhibitors or ARBs; the ORs (95% CI) were 0.99 (0.81–1.21) and 0.96 (0.72–1.28), respectively. As compared with ARBS, the OR for ACE inhibitors was 1.00 (0.71–1.40). In all subgroups and sensitivity analyses, there was no association of pneumonia with use of ACE inhibitors or ARBs in relation to stroke, diabetes, advanced age, or study setting. Thus, we conclude that our study results are robust.

**Table 2.** Association of pneumonia with ACE inhibitor and ARB use

		Crude OR			Adjusted OR^a^		
			
		OR	95% CI	*P* value	OR	95% CI	*P* value
General population	ACE inhibitors	1.85	(1.56, 2.19)	<0.0001*	0.99	(0.81, 1.21)	0.90
	ARBs	1.59	(1.24, 2.03)	0.0002*	0.96	(0.72, 1.28)	0.77

Stroke	ACE inhibitors	1.11	(0.59, 2.11)	0.74	0.85	(0.44, 1.65)	0.62
	ARBs	1.47	(0.66, 3.28)	0.34	1.24	(0.54, 2.82)	0.61

Diabetes	ACE inhibitors	1.88	(1.37, 2.58)	<0.0001*	1.12	(0.78, 1.60)	0.54
	ARBs	1.30	(0.86, 1.97)	0.21	0.98	(0.62, 1.55)	0.94

Age ≥65 yrs	ACE inhibitors	1.93	(1.58, 2.37)	<0.0001*	1.17	(0.93, 1.47)	0.18
	ARBs	1.62	(1.21, 2.16)	0.001*	1.08	(0.78, 1.49)	0.66
**Sensitivity analysis**							
Index date moved to 14 days before admission	ACE inhibitors	1.23	(1.03, 1.47)	0.02*	1.06	(0.89, 1.28)	0.51
	ARBs	1.37	(1.06, 1.77)	0.02*	1.24	(0.95, 1.6)	0.11

Time period: 14 days	ACE inhibitors	1.84	(1.55, 2.19)	<0.0001*	0.98	(0.79, 1.22)	0.87
	ARBs	1.65	(1.29, 2.11)	<0.0001*	1.06	(0.79, 1.42)	0.70

Time period: 60 days	ACE inhibitors	1.92	(1.62, 2.27)	<0.0001*	1.08	(0.89, 1.3)	0.43
	ARBs	1.58	(1.23, 2.02)	0.0003*	0.97	(0.74, 1.27)	0.82

Time period: 75 days	ACE inhibitors	2.00	(1.69, 2.37)	<0.0001*	1.19	(0.98, 1.43)	0.07
	ARBs	1.60	(1.25, 2.05)	0.0002*	0.99	(0.75, 1.3)	0.93

Abbreviations: ACE, angiotensin-converting enzyme; ARB, angiotensin II receptor blocker.

^a^Adjusted for use of ACE inhibitors, ARBs, statins, histamine type-2 antagonists, proton-pump inhibitors, and outpatient clinic visits.

*P < 0.05.

The associations between drug dose and pneumonia are shown in Table 3. No significant association with pneumonia for any cumulative DDD (ie, 0 to 30, 31 to 60, or >60 DDDs) as compared with nonusers. The ORs (95% CI) were 0.94 (0.76–1.17), 1.23 (0.88–1.71), and 0.88 (0.5–1.56), respectively, for ACE inhibitors and 0.95 (0.71–1.27), 0.95 (0.63–1.43), and 1.92 (0.73–5.03), respectively, for ARBs. There was no dose–response trend in the principal or subgroup analyses. All the *P* values for trends were greater than 0.05, and the results were robust in sensitivity analyses.

**Table 3.** Association of pneumonia with ACEI and ARB dose

	DDD	ACEI^a^			ARB^a^		
		
		OR	95% CI	*P* for trend	OR	95% CI	*P* for trend
General population	0	1.00			1.00		
	1–30	0.94	(0.76, 1.17)	0.80	0.95	(0.71, 1.27)	0.86
	31–60	1.23	(0.88, 1.71)		0.95	(0.63, 1.43)	
	>60	0.88	(0.50, 1.56)		1.92	(0.73, 5.03)	

Stroke	0	1.00			1.00		
	1–30	0.77	(0.37, 1.61)	0.98	1.24	(0.54, 2.84)	0.92
	31–60	0.95	(0.40, 2.27)		1.17	(0.38, 3.60)	
	>60	1.28	(0.26, 6.16)		0.44	(0.03, 6.94)	

Diabetes	0	1.00			1.00		
	1–30	0.98	(0.67, 1.43)	0.17	1.02	(0.64, 1.62)	0.65
	31–60	1.81	(1.02, 3.23)		0.76	(0.41, 1.42)	
	>60	1.43	(0.56, 3.69)		1.46	(0.38, 5.61)	

Age ≥65 yrs	0	1.00			1.00		
	1–30	1.08	(0.85, 1.38)	0.09	1.065	(0.77, 1.48)	0.53
	31–60	1.56	(1.09, 2.25)		1.034	(0.65, 1.64)	
	>60	1.15	(0.62, 2.10)		2.207	(0.73, 6.65)	

Abbreviations: ACE, angiotensin-converting enzyme; ARB, angiotensin II receptor blocker.

^a^Adjusted for use of ACE inhibitors, ARBs, statins, histamine type-2 antagonists, proton-pump inhibitors, and outpatient clinic visits.

*P < 0.05.

## DISCUSSION

We found no significant association between pneumonia requiring hospitalization and use of ACE inhibitors or ARBs in the Taiwanese general population, and ACE inhibitors and ARBs had a similar null effect on pneumonia risk. We also found no dose–response relationship between cumulative DDD and pneumonia. In subgroup analyses, there was no significant association of pneumonia requiring hospitalization with ACE inhibitor use, ARB use, or cumulative DDD among patients with stroke or diabetes or among elderly adults.

By using a case-crossover design, we were able to control for time-invariant between-person confounding factors, and our findings were consistent with those of previous studies, which showed no protective effect of ACE inhibitor use on pneumonia requiring hospitalization in a general population or among patients with coronary disease.^13^^,^^14^ A difference between ACE inhibitors and ARBs is that ACE inhibitors but not ARBs increase the level of substance P and improve symptomless dysphagia.^28^ We also investigated if the effects of ACE inhibitors and ARBs differed in a general population. We enrolled patients with a first episode of pneumonia requiring hospitalization. They were relatively young (mean age, 57 years) and had less impairment in cough reflex (<5% were stroke patients). Hence, differences between ACE inhibitors and ARBs were not obvious.

Previous studies showed that ACE inhibitors can prevent aspiration pneumonia among elderly stroke patients.^6^^–^^11^ One international clinical trial of ACE inhibitor use among stroke patients showed that ACE inhibitor use had a preventive effect on pneumonia only in Asian populations.^12^ Because stroke patients may have impaired cough reflex and are more likely to be hospitalized for aspiration pneumonia, we examined the effects of ACE inhibitors on pneumonia risk among patients with a history of stroke. We found that use of ACE inhibitors was associated with a decrease in pneumonia risk (ORs = 0.85; 95% CI = 0.44–1.65); however, due to the small number of cases (*n* = 527), the finding was not statistically significant. This result is consistent with the findings of a recent report.^11^ We also examined if the effect of ARBs differed from those of ACE inhibitors among stroke patients. However, the results were inconclusive due to the small number of stroke patients in the analysis.

We conducted a subgroup analysis of elderly adults because of the higher incidence of silent aspiration among elderly patients with community-acquired pneumonia.^2^ The 2009 Japanese Society of Hypertension (JSH) Guidelines for the Management of Hypertension specify the use of ACE inhibitors for hypertensive patients with repeated aspiration pneumonia, including silent aspiration.^29^ In our subgroup analysis, there was no significant association between ACE inhibitor use and pneumonia among elderly adults. Our study population including patients with a first episode of pneumonia requiring hospitalization. They were relative healthy and had an unimpaired cough reflex, which might explain why we did not observe a protective effect among this population.

A previous study reported a significant protective effect of ACE inhibitors on outpatient pneumonia among people with diabetes and a general population.^15^^,^^16^ In addition, a dose–effect relationship was reported between ACE inhibitor use and outpatient pneumonia in diabetes patients.^15^ We selected pneumonia requiring hospitalization, but not outpatient pneumonia, as an outcome variable, because discharge diagnoses codes are more accurate than outpatient coding. Perhaps this difference in outcome variable (pneumonia requiring hospitalization vs outpatient pneumonia) is a reason for the difference between the present and previous results.

A recent comprehensive review found that ACE inhibitors but not ARBs had a protective effect against pneumonia.^30^ However, two important points should be considered in interpreting the difference between our findings and those of the systematic review and meta-analysis. First, the participants of the studies included in the systematic review were older than our patients. Older adults have a higher prevalence of dysphagia, including silent aspiration. Hence, the protective effects of ACE inhibitors, via increased cough reflex, were easier to observe. Second, most studies included in the review investigated people with a history or risk of cerebral vascular disease. As we previously reported, ACE inhibitors reduced pneumonia risk among post-stroke patients.^11^ Thus, the pooled results of those studies showed that ACE inhibitors had a protective effect.

In this study, we included a general population with first pneumonia requiring hospitalization. They were relative young (mean age 57 years) and had less comorbidity (history of stroke: 4.8%). Hence, the protective effects of ACE inhibitors were not obvious. In our judgment, ACE inhibitors may have a preventive effect on pneumonia in certain populations, such as people with impaired cough reflex. However, the effect of ACE inhibitors on pneumonia was not significant among a general population.

We used a case-crossover design for the present study. This design was first used to study transient exposures and acute outcomes, but was it recently applied to prolonged drug exposures and longer-term outcomes.^31^ In sensitivity analysis, our results remained robust for different time periods, even when a 75-day time period was evaluated.

We performed additional adjusted analyses of respiratory medication—namely, xanthines: R03DA; glucocorticoids (inhalants): R03BA; anticholinergics (inhalants): R03BB, β-adrenoreceptor agonists (inhalants): R03AB, R03AC; β-adrenoreceptor agonists: R03CB, R03CC; leukotriene antagonists: R03DC; and glucocorticoids: H02AB—as time-variant confounding factors, and the results were unchanged (data not shown). We also performed subgroup analyses of patients with respiratory disease (patients using respiratory medication during the case or control period), and the results were unchanged (data not shown).

This report describes a nationwide population study. To our knowledge, this is the first analysis of nationwide administrative data on this topic among an Asian population, which is of considerable importance, as Asian populations are considered to be more susceptible to the effects of ACE inhibitors on cough reflex. Our findings likely reflect the real-world effects of the studied drugs on Asian people. This design permitted auto-matching for unmeasured time-invariant confounding factors, which results in a clearer comparison.

There were some limitations in the present study. First, we had no data on smoking, lifestyle, functional status, cognitive status, or the severity of lung disease, heart disease, or impairment in cough reflex. However, these factors are unlikely to change substantially during a short period of time and are thus controlled for in a case-crossover design. Second, we only included cases of pneumonia requiring hospitalization. However, not all pneumonia patients are treated as inpatients. Our analysis would be unable show that ACE inhibitors or ARBs have a protective effect only among outpatients. Third, the study population comprised adults who were hospitalized for pneumonia for the first time. Therefore, they were relative healthy and had no impairment in cough reflex. If the protective effect of ACE inhibitors were limited to frail people, we would be unable to observe it. Finally, the case-crossover design might not be appropriate if all patients carefully adhered to treatment with ACE inhibitors and ARBs. Persistent use of ACE inhibitors and ARBs would reduce variability of exposure status during the case and control periods and would bias our results toward the null. However, real-world adherence to drug therapy for chronic diseases is usually not good.

Our study showed no significant association of pneumonia requiring hospitalization with use of ACE inhibitors or ARBs among the Taiwanese general population. In addition, there was no dose–response effect of ACE inhibitors or ARBs on pneumonia requiring hospitalization.
